# Serum leptin level as a diagnostic and prognostic marker in infectious diseases and sepsis: A comprehensive literature review

**DOI:** 10.1097/MD.0000000000025720

**Published:** 2021-04-30

**Authors:** Victoria Birlutiu, Loredana Camelia Boicean

**Affiliations:** a “Lucian Blaga” University of Sibiu, Faculty of Medicine; b Academic Emergency Hospital Sibiu—Infectious Diseases Clinic, Sibiu, Romania.

**Keywords:** hyperleptinemia, infectious diseases, leptin deficiency, leptin resistance, sepsis

## Abstract

**Background:**

Infections and sepsis are common causes of morbidity and mortality, with an increasing incidence worldwide. Leptin is involved in the inflammatory process and may modulate the cytokine production, immune cell proliferation and endothelial function. There are conflicting results regarding alterations of leptin levels in infectious diseases and the outcome from sepsis.

The aim of the current article is to provide an overview of the medical literature on the correlations between variations of leptin levels and infectious diseases and sepsis.

**Methods:**

We performed an extensive literature search in PubMed and Google Scholar databases, using keywords to identify articles related to leptin in infectious diseases and sepsis. Searches were referenced using medical subject headings that included “leptin,” “adipokines,” “sepsis,” “infectious diseases,” “leptin deficiency,” “leptin resistance” or “hyperleptinemia.” The language of publication, journal, or country were not included as limitation criteria.

Articles or abstracts containing adequate information, such as age, sex, anthropometric indices, clinical presentation, comorbidities, and management were included in the study, whereas articles with insufficient clinical and demographic data were excluded. We assessed the quality of the studies selected.

The final review of all databases was conducted on June 18, 2020.

**Results:**

We find the results from the current review to be of great importance due to the possible therapeutic role of leptin analogs in states of leptin deficiency associated with infectious diseases or sepsis.

In hyperleptinemia, a therapeutic plan for obtaining leptin neutralization also needs further investigations. This could lead to the reduction of proinflammatory responses.

There is a need for further studies to demonstrate the specificity and sensitivity of leptin in the early diagnosis of sepsis and the need to measure serum leptin levels in routine evaluation of the critical patient.

**Conclusion:**

The multiple effects of leptin are of growing interest, but further studies are needed to elucidate the role of leptin signalling in infectious diseases and sepsis. Because very few human studies are reported, we recommend the need for further research.

Better understanding of the pathophysiology of sepsis and the implication of circulating total leptin in this process could help physicians in managing this life-threatening condition.

## 1 Introduction

Infections and sepsis are common causes of morbidity and mortality, with an increasing incidence worldwide. Sepsis is a life-threatening condition and an adequate management is decisive to overcome sepsis-associated mortality.^[1,2]^

Leptin is involved in the inflammatory process and may modulate the cytokine production, immune cell proliferation, and endothelial function. There are conflicting results regarding alterations of leptin levels and the outcome from sepsis.^[3,4]^

The aim of the current article was to provide an overview of the medical literature on the correlations between variations of leptin levels and infectious diseases and sepsis.

Leptin deficiency and leptin resistance are studied as pathogenic factors in bacterial, viral, and parasitic infections, with effect on cytokine production, increased susceptibility to infections and altered inflammatory response, and hyperleptinemia is considered an independent predictable factor in the development of sepsis and an unfavourable outcome.^[3,5–8]^

## 2 Methods

We performed an extensive literature search in PubMed and Google Scholar databases, using keywords to identify articles related to leptin in infectious diseases and sepsis. Searches were referenced using medical subject headings that included “leptin,” “adipokines,” “sepsis,” “infectious diseases,” “leptin deficiency,” “leptin resistance,” or “hyperleptinemia.” The language of publication, journal or country were not included as limitation criteria.

Articles or abstracts containing adequate information, such as age, sex, anthropometric indices, clinical presentation, comorbidities, and management were included in the study, whereas articles with insufficient clinical and demographic data were excluded. We assessed the quality of the studies selected.

The final review of all databases was conducted on June 18, 2020.

## 3 Results

This review aims to evaluate the role of serum leptin in the early diagnosis of sepsis and as a predictive factor of its evolution. There were significant findings that we observed while conducting this literature review.

The nutritional status is essential for an adequate immune response in infections^.^^[1]^ Obese patients have increased leptin levels because of hyperleptinemia or leptin resistance and in malnutrition patients present leptin deficiency. Aberrant leptin levels may induce an altered inflammatory response and increased susceptibility to bacterial, viral, and parasitic infections. Hyperleptinemia is considered a predictable factor in the development of sepsis and of an unfavourable outcome.^[1,8–12]^

There are studies that emphasize the importance of monitoring serum leptin levels in critically ill patients to distinguish between sepsis and non-infectious systemic inflammatory response syndrome (SIRS).^[9,13]^ One of them showed a correlation between leptin, interleukin-6 (IL-6) and tumour necrosis factor-α (TNF-α) in sepsis. The authors found out that a serum leptin threshold of 38 μg/L can differentiate sepsis from noninfectious SIRS, with a sensitivity of 91.2% and a specificity of 85%.^[9]^

There is an increase in leptin levels in patients with sepsis and a decrease in leptin levels in patients with an unfavourable outcome. Higher leptin levels in patients who survived from acute sepsis were reported in literature. A study reveales that leptin levels above 10 ng/mL were significantly associated with in-hospital mortality.^[3]^ Low leptin and high IL-6 levels indicated an unfavourable prognosis in patients with sepsis, probably because of the absence of the leptin's protective effect.^[10]^

Experimental studies reveal that leptin-deficient and leptin receptor-deficient mice present thymic atrophy and a defective immune response.^[1]^ Reduced leptin levels in individuals with malnutrition were also associated with altered immune functions and thymic atrophy. These conditions can be reversed by exogenous leptin administration according to the results published in different studies.^[1,14–17]^

We find the results from the current review to be of great importance due to the possible therapeutic role of leptin analogs in infectious diseases and sepsis.

In hyperleptinemia, a therapeutic plan for obtaining leptin neutralization also needs further investigations. This could lead to the reduction of proinflammatory responses in patients with obesity.

Because few human studies have been reported in literature, there is a need for further studies to demonstrate the specificity and sensitivity of leptin in the early diagnosis of sepsis and the need to measure serum leptin levels in routine evaluation of the critical patient.

## 4 Discussion

### 4.1 Leptin

Leptin, a hormone mainly generated by adipocytes, mediates the relationship between food intake and energy expenditure, has a role in body mass control, metabolism and endocrine functions, in the immune response and homeostasis, in the development and functioning of the lung tissue, in reproduction and in the onset of puberty.^[18–20]^ There are other cells that secrete leptin under certain conditions, such as those in the pituitary gland and hypothalamus,^[21]^ placenta,^[22]^ mammary epithelium,^[23]^ lung epithelium, bronchial tissue, type II pneumocytes and lipofibroblasts, stomach,^[24]^ colon, skeletal muscle,^[25,26]^ and bone.^[18]^

Leptin is a polypeptide and it belongs structurally to the cytokine family. It is encoded by the Ob gene, located on the long arm of chromosome 7, at the position 31.3 (7q31.3), has about 20 Kb length and its receptors have an almost ubiquitary distribution, thus causing multiple biological effects.^[8,27,28]^

Leptin has a role in both innate and adaptive immunity, it acts as a proinflammatory cytokine and its concentration is influenced by the body mass index, hormones and gender, women having higher concentrations of total circulating leptin compared to men.^[1,29,30]^

Leptin synthesis is regulated by food intake and correlates with insulin levels. It is increased by glucocorticoids and ovarian sex steroids, and inhibited by testosterone.^[31–33]^ Proinflammatory cytokines acutely increase leptin expression, but chronic stimulation with proinflammatory cytokines suppress leptin production.^[34–37]^

Leptin deficiency and leptin resistance induce alterations in cytokine production and increase the susceptibility towards infectious diseases.^[8]^ There are studies that associate the effects of an increased body mass index, which induces leptin resistance, as well as the effects of malnutrition, which induces leptin deficiency, with the likelihood of developing bacterial and viral lung infections, and also parasitic infections.^[1,38]^

### 4.2 The mechanism of action of leptin

Leptin circulates freely in the blood stream and its actions take place using membrane bound leptin receptors. Leptin receptor gene (Ob*-*R) includes short isoforms (Ob*-*Ra, Rc, Rd, and Rf), 1 long isoform (Ob-Rb), and 1 soluble isoform (Ob-Re).^[28,39]^ Leptin acts via a transmembrane receptor, which is structural related with the class I cytokine receptor superfamily. Class I cytokine receptor superfamily is characterized by extracellular domains.^[8,39,40]^

The primary signalling route is Janus kinase/signal transducer and activator of transcription (JAK/STAT) pathway.^[41–43]^ JAK2 activation induces phosphorylation of tyrosine-based motifs, which act as binding sites for signalling molecules as STAT-1, STAT-3, STAT-5, and STAT6. Other alternative pathways can be induced by leptin, including the phosphoinositide 3-kinases/ phosphodiesterase 3B/cyclic adenosine monophosphate pathway, adenosine monophosphate-activated protein kinase, mammalian target of rapamycin (mTOR), and the mitogen-activated protein kinases cascade.^[8,20,42,44]^ Leptin signalling is inhibited by the increased expression of SOCS-3, which affects JAK/STAT pathway by binding to the Ob-Rb and induces dephosphorylation of JAK2.^[28,45]^ There is also data in literature according to which changes in SOCS-3 expression are related to leptin resistance found in obesity. A negative feedback regulator of Ob-R signalling is protein tyrosine phosphatases 1B (PTP1B), which determines dephosphorylation of JAK2.^[8,20,28,44,46–49]^

The central function of leptin is metabolic homeostasis, by providing information about the total body fat mass to the hypothalamus. The hypothalamus responds by regulating glucocorticoids and insulin, food intake, and energy balance.^[1]^ Leptin acts via specific receptors in the hypothalamus to control food intake and to permit energy expenditure and appropriate glucose homeostasis.^[50,51]^ Leptin activates a neural circuit which includes anorexigenic and orexigenic neuropeptides to regulate appetite.^[51]^ Orexigenic peptides include neuropeptide Y, melanin-concentrating hormone, agouti-related protein (AgRP), galanin, orexin, and galanin-like peptide. Anorexigenic peptides include pro-opiomelanocortin (POMC), cocaine-regulated transcript and amphetamine-regulated transcript, neurotensin, corticotropin-releasing hormone, and brain-derived neurotrophic factor.^[52]^ Long-term leptin administration in leptin-deficient mice has been shown to increase the number of synapses on neurons that produce POMC and to reduce the number of synapses on neurons that produce Neuropeptide Y.^[51,53]^ Leptin activates POMC neurons, which secrete α-melanocyte-stimulating hormone. α-melanocyte-stimulating hormone activates the melanocortin-4 receptor to induce satiety and increase energy use. Leptin also inhibits AgRP neurons, promoting feeding and reducing energy expenditure. These facts suggest that POMC and AgRP neurons are essential for leptin action and for energy homeostasis.^[50]^

Leptin also interacts with the mesolimbic dopamine system, which is involved in the interest for feeding, and with the nucleus of the solitary tract, which controls satiety.^[51]^

Leptin regulates glucose metabolism in the liver through leptin receptors and indirectly through the hypothalamic receptors. Leptin increases glycogen synthesis and storage, suppresses gluconeogenesis, and prevents glucose oxidation by reducing the metabolism of glucose in peripheral tissues.^[54]^

High leptin levels increase the energy use proportional to body fat and decreases food intake. “Leptin may increase energy expenditure by triggering the uncoupling protein action.”^[54]^

Fasting and prolonged caloric restriction have been shown to result in a decrease in plasma leptin concentration and this induces a neuroendocrine response, reducing the energy use and promoting an orexigenic signal. It also reduces hepatic insulin sensitivity and increases hepatic glucose production.^[50,51]^ Low leptin levels also induce a decrease in sexsteroids levels, a decrease in thyroid hormone levels that will reduce the metabolic rate, a decrease in insulin-like growth factor-1 levels that will slow growth-related processes, and an increase in growth hormone levels that will mobilize energy stores. These results were obtained in both mice and human studies.^[51,55–57]^

Due to the structural similarities with members from cytokine family, leptin acts as a cytokine, having a key role in immunity, hematopoiesis and angiogenesis. Because it is derived from adipose tissue, it is also called “adipokine,” together with adiponectin, apelin, chemerin, and others.^[28]^

Leptin functions as a hormone and as a regulator of immunity, acting as a proinflammatory cytokine-like IL-1, IL-6, IL-8 and TNF-α. In this way, leptin deficiency increases the susceptibility to infections.^[1,38,58]^ Leptin has a proinflammatory immune response in lungs, stimulating neutrophil and macrophage chemotaxis and increasing oxidative stress, phagocytosis, and cytokine production.^[8,20,44,59–62]^ Leptin induces T-lymphocytes proliferation, has antiapoptotic effects on them and increases T helper cell differentiation.^[8,20,44,63–65]^ Leptin regulates both innate and acquired immunity, increases the immune response by mediating the production of proinflammatory cytokines and by activating Th cells, leading to the activation of monocytes and macrophages and preventing the apoptosis of various immune cells.^[1]^ In innate immunity, leptin amplifies the function of neutrophils, by releasing oxygen free radicals, and the cluster of differentiation molecule 11B (CD11b) expression, leading to the migration of immune cells at the site of inflammation. It also activates monocytes and dendrite cells, thus increasing the secretion of proinflammatory cytokines such as TNF-α, IL-6 and IL-12, leading to the activation of natural killer (NK) cells.^[1,66,67]^ In adaptive immunity, leptin reduces the rate of apoptosis of thymic T-cells by inhibiting FAS-directed apoptosis pathway, but also by up-regulating the expression of B-cell lymphoma-extra large and T-box transcription factor, inducing the maturation of thymic T-cells.^[1,68–70]^

“Leptin deficiency, a reduced leptin signalling and an increased expression of SOCS-3, all reported in obesity, hypertriglyceridemia, insulin resistance and malnutrition, represent risk factors in host immunity.” “Viral infections cause elevated SOCS-3 expression, which inhibits leptin signalling.” It results in immunosuppression by T-regulatory cells and the host defence system fails to manage the aggression of pathogens, inducing infections and diminished vaccine-specific antibody response.^[28]^

Alti and colleagues presented the intervention of leptin in infections and they proposed leptin as an adjuvant tool in the development of new vaccines. Administration of exogenous leptin was used with promising results as vaccine against *Rhodococcus equi* and *Helicobacter pylori* in mice. Leptin signaling in gut cells offered protection against *Clostridium difficile* (*C difficile*) and *Entamoeba histolytica* infections.^[28]^

### 4.3 Plasma leptin levels in inflammation and respiratory infections

There are studies on mice that demonstrated that leptin has a role in the respiratory function. Tachypnea, hypoventilation, and respiratory depression are more frequent in cases of obesity and seem to be attenuated by long-term leptin administration.^[71,72]^ These findings suggest that leptin may be a modulator of central respiration.

Malnutrition, which is associated with reduced leptin levels, and obesity, which is associated with leptin resistance, increase the susceptibility to bacterial or viral respiratory infection, such as the H1N1 flu epidemic.^[1,38,73–78]^

Diez and colleagues studied the implications of leptin levels in community acquired pneumonia. They suggest that leptin does not act as an inflammatory reactant but as a nutritional marker in community acquired pneumonia.^[79]^

Parmentier and colleagues showed in their study that serum leptin levels were not associated with ventilator-associated pneumonia, but they emphasize the need for further studies to confirm their results and to define the inflammatory role of leptin as a biomarker in intensive care unit patients.^[80]^

### 4.4 Leptin in bacterial infections

Some studies showed that in leptin-deficient mice, there are alterations in macrophage- and neutrophil-mediated phagocytosis of bacteria.^[8,81–84]^ Macrophages also showed impaired leukotriene synthesis in vitro and alterations in leukotriene synthesis has been found in patients predisposed to pulmonary infections. All these pathological changes were demonstrated to be restored by the administration of leptin in leptin-deficient mice.^[8,82,85–87]^ Signalling routes STAT3 and ERK play an essential role in host defence against bacterial pneumonia.^[82,86,87]^

Mice infected with *Klebsiella pneumoniae* (*K pneumoniae*) showed high leptin levels. It remains to be studied if this is because of the increased synthesis of leptin in the lungs or because of the leakage of leptin from the circulation, both present in pulmonary inflammation.^[8,20,44,82,86,87]^ Leptin deficient mice showed a low survival rate after *K pneumoniae* infection*, Streptococcus pneumoniae* (*S pneumoniae*) and *Mycobacterium abscessus* (*M abscessus*). This demonstrates that leptin has a significant role in the immune response against bacterial aggressions.^[8,82,83,86–88]^

### 4.5 Leptin in viral infections

There is some data reported in literature on the effects of hyperleptinemia, which leads to leptin resistance, on the immune response to viral infections. An inadequate immune response, with alterations in NK-cell cytotoxicity and delayed proinflammatory cytokine response were found in diet-induced obese mice, which also presented a higher mortality rate upon viral infections.^[8,20,44,89,90]^ Because of the peripheral leptin resistance which leads to impairment in the IL-15 function, diet-induced obesity can affect the T-cell populations in the lung tissue.^[8,20,44,91,92]^

In diet-induced obesity and leptin deficiency in experimental models with influenza infection there are alterations in mononuclear cells and dendritic cells functions, with consequences on the immune system.^[8,44,91–94]^

Morgan and Azzoni reported that impaired leptin signalling is caused by an altered defence response in host for influenza AH1N1 and human immunodeficiency virus (HIV) infections, due to the alterations of the immunologic, metabolic, and endocrine processes.^[28,95,96]^

The findings of Zhang et al showed that leptin has no implication in viral replication, but high levels of serum leptin are associated with severe lung injury by AH1N1 in diet-induced obesity in mice.^[27]^ Low leptin levels in patients with obesity suppress the function of memory T-cell, Interferon (IFN)-α, IFN-β, and IFN-γ and could increase the susceptibility to influenza virus infections.^[1]^

Patients with HIV infection have an exaggerated expression of leptin receptors and exogenous leptin administration in these patients may reduce their metabolic complications. Oxidative status of monocytes, which leads to an altered immune response in HIV infection, may be diminished by leptin analogs.^[1]^

### 4.6 Leptin resistance and hyperleptinemia

Only a few studies reported the effects of high leptin levels and leptin resistance secondary to obesity on the immune response to viral infections and have shown an increase in the mortality rate.^[1,3,8,9,20,44,96]^

The prevalence of obesity is increasing and obesity is frequent in patients with sepsis.^[97,98]^ The adipose tissue, through the production of cytokines and adipokines, with vasoactive effect, is considered a modulator of inflammation and immunity.^[82,83]^ Adipokines modulate cytokine production, immune cell proliferation, and endothelial function, having a key role in the inflammatory process.^[65,99]^

In a study published by Jacobsson and colleagues, which was the first prospective study to determine total serum levels of leptin and adiponectin in patients with sepsis, high levels of circulating leptin at baseline were reported to be predictors of sepsis and leptin predicted sepsis-related in-hospital death rate. High levels of circulating leptin were correlated with a first sepsis event and there was a stronger association between high levels of leptin at baseline and more severe forms of sepsis.^[1]^ Jacobsson and colleagues found out that leptin levels above 10 ng/mL were significantly associated with in-hospital mortality.^[3]^

Baseline leptin levels reflect the nutritional status and correlate to the amount of fat mass.^[99–101]^ High levels of leptin have a proinflammatory effect, inducing the activation of the innate immune system and the release of proinflammatory cytokines. The systemic inflammation and vasoplegia in sepsis lead to hypotension and septic shock. Hyperleptinemia in obese patients induces alterations in endothelial functions and determines an unfavourable evolution or septic shock.^[3,102–104]^ Experimentally, diet-induced obesity in mice leads to a chronic increase in leptin and leptin resistance, inducing a modified immune response, including low cytotoxicity of NK cells and delayed expression of proinflammatory cytokines.^[1,8,20,44]^

### 4.7 Leptin deficiency

Reduced serum leptin levels can induce malnutrition and a state of immunodeficiency.^[1]^ Leptin deficiency in both mouse and human studies induces an altered immune response. The alterations in the immune responses are produced mainly by a decrease in total lymphocytes and CD4+ helper T cells number, and also by thymic cells apoptosis, resulting an increased susceptibility to infections.^[1]^

In leptin receptor-deficient mice, studies showed that STAT3, STAT5, and ERK pathways play a key role in host defence against bacterial infections and in leukocyte function.^[1,8,16,20,44,105]^

Experimentally, mice with leptin deficiency or leptin receptor deficiency have been shown to have thymus atrophy and poor immune response. Similarly, in patients with malnutrition, in whom leptin deficiency is present, the same changes occur and these are reversible to the administration of exogenous leptin, emphasizing the possible therapeutic role of leptin analogues in the control of infectious diseases.^[1,8]^

Studies reported that leptin deficiency is correlated with nutritional deprivation or malnutrition, which affects the innate and acquired immunity of the host, by decreasing the number of T lymphocytes, CD4 + and CD8 +, thus increasing the incidence of infections and the mortality rate. The leptin deficiency present in malnutrition is correlated, through the deficient production of cytokines, with bacterial, viral and parasitic infections, such as pneumonia, flu, tuberculosis, sepsis, colitis, leishmaniasis, trypanosomiasis, amoebiasis, and malaria.^[1,8,27,37,106–109]^

Leukotrienes have been shown to increase macrophage-mediated phagocytosis and poor leukotriene synthesis has been found in some patients who have been shown to be susceptible to lung infections. Studies showed that leptin-deficient mice have poor survival rate after administration of*K pneumoniae*, *S pneumoniae,* and *M abscessus*, which does not appear to be due to impaired inflammatory cell recruitment but rather as a result of defective phagocytosis. Moreover, macrophages in leptin deficient mice showed decreased in vitro leukotriene synthesis. Mice infected intratracheally with *K pneumoniae* have elevated serum leptin levels. The macrophages- and neutrophils-mediated phagocytic response and the leukotrienes production can be restored by administration of leptin to leptin-deficient mice.^[8,20,44]^

Recent observational and interventional studies in humans showed that leptin replacement therapy could improve the management in leptin deficiency syndromes. “R-metHuLeptin replacement therapy could prove to be a new potentially useful medication for states of absolute or relative leptin deficiency to restore neuroendocrine, metabolic or immune function in low-leptin states such as (congenital or acquired) lipodystrophy or exercise-induced hypothalamic amenorrhea.” To prove the efficacy of leptin analogs more long-term interventional studies are needed.^[110]^ Leptin replacement therapy in pediatric patients with congenital leptin-deficiency restored the Th1/Th2 cytokine balance and induced proliferation of lymphocytes, neutrophils and monocytes, with no effect on body weight.^[17,28]^

### 4.8 Serum leptin levels in critically ill patients

Leptin is recognized for its role in the neuroendocrine response to stress, but also for its role in human defence mechanisms. In sepsis, the neuroendocrine and metabolic responses take place in an acute first phase, by activating the hypothalamic-pituitary-adrenocortical axis, with hypersecretion of growth hormone in the presence of low concentrations of insulin-like growth factor-1 and thyroid hormones, as well as low gonadal activity. Insulin resistance and a decreased glucose use appear, beneficial in reducing the energy consumption of the macroorganism. Systemic inflammation, insulin resistance and hyperglycemia are common features of critical illness.^[111–114]^ In cases where this stage lasts more than 8 to 10 days, there is an inhibition of hypothalamic-pituitary function with the development of cachexia, even under conditions of a normal diet of the patient. The role of leptin has not been much investigated in sepsis, but the administration of endotoxin to healthy volunteers increases its concentration at much higher levels compared to control groups.^[112–114]^

Sepsis is defined, according to 2016 terminology, by life-threatening organic dysfunction caused by a defective response of the host to infection. “Sequential [Sepsis-related] Organ Failure Assessment” (SOFA) score is a marker for organ dysfunction and disease severity. Organic dysfunction is assessed based on an increasing SOFA score by 2 points or more, associated with in-hospital mortality greater than 10%.^[3,115]^ The occurrence of circulatory, cellular, and metabolic abnormalities, which increase the risk of in-hospital mortality by over 40%, defines septic shock. Hypotension is associated with persistent sepsis, despite adequate volume rebalancing, which requires positive inotropic support.^[115–117]^

Monitoring circulating total leptin levels has been proven to have an important role in distinguishing sepsis from SIRS with an etiology other than infection. Yousef and colleagues found out that a serum leptin cut-off level of 38 μg/L has a key role in differentiation between sepsis and noninfectious SIRS, with a sensitivity of 91.2% and specificity of 85%.^[9]^

In a study conducted by Bornstein and colleagues, it was revealed that there is a loss of diurnal rhythm in cortisol and leptin secretion in patients who survived acute sepsis. In the control group serum leptin and cortisol levels had circadian rhythms with increased nocturnal total serum leptin levels and low nocturnal cortisol levels. “Mean leptin levels were three-fold higher in patients who survived the septic episode than in non-survivors.”^[10]^ Similar results were published, suggesting that hyperleptinemia may be a host defense mechanism during sepsis.^[11]^

Tzanela and colleagues showed that leptin levels are higher at the onset of sepsis and they correlate with insulin levels and insulin resistance. Low leptin levels were found in prolonged sepsis, but findings revealed that this was not related to survival.^[12]^

Severe hypotension, tachycardia, and immune response increase are observed in sepsis. Sepsis is responsible for multiple organ dysfunction and high mortality rate. Leptin administration has a protective role against the effects of sepsis and endotoxemia. It is has been suggested that leptin elevates blood pressure.^[21,58,118]^ “Vallejos and colleagues reported that leptin administration reduced hypotension and tachycardia on experimental models.” Their study also reported that leptin administration decreased the oxidative stress and reduced proinflammatory cytokines levels, such as TNF-α, IL-1β, and IL-6. They demonstrated that administration of exogenous leptin decreased the death risk in sepsis at early and late stages. It is taken into consideration the potential preventive therapy with exogenous recombinant leptin against sepsis in critically ill patients.^[118]^

There is some data published that shows that alteration of the STAT3 signalling pathway increases susceptibility to *C difficile* infectious colitis and bacterial peritonitis, and leptin administration has been shown to restore the protective response of the intestinal mucosa in *C difficile* infection in both mice and humans.^[1,109]^

Takahashi and colleagues report that leptin deficiency is fatal in mice suffering from sepsis due to multiple organ failure.^[119]^ Systemic leptin replacement modulated the immune response against sepsis and increased survival in leptin-deficient mice. Tschöp and colleagues reported higher leptin levels in patients recovered from sepsis compared to that of nonsurvivors. These results also suggest the potential therapeutic benefits of leptin in infectious disease.^[28,120]^

## 5 Conclusions

Over the past decade, the role of total serum leptin in infectious diseases and sepsis has been explored in experimental models on mice and in few human studies.

This review shows results from different studies. A significant progress has been made in understanding the role of leptin in the immune response and in inflammatory and infectious diseases. Emerging data from animal models and humans indicates that leptin could control the systemic immune defense response and further suggests the possible therapeutic potential of exogenous leptin administration in infectious diseases and sepsis. Monitoring serum leptin levels in critically ill patients could be of great value for early diagnosis and prognosis of sepsis. The early diagnosis of sepsis, the identification of its origin and an adequate therapeutic management are crucial to overcome sepsis-associated mortality.

The multiple effects of leptin are of growing interest, but further studies are needed to elucidate the role of leptin signalling in infectious diseases and sepsis. This could contribute to new therapeutic approaches and a more favourable outcome in this pathology. Because most of the studies regarding this subject are based on animal models and very few human studies are reported, we recommend the need for further research.

## Author contributions

**Conceptualization:** Victoria Birlutiu, Loredana Camelia Boicean.

**Data curation:** Victoria Birlutiu, Loredana Camelia Boicean.

**Formal analysis:** Victoria Birlutiu, Loredana Camelia Boicean.

**Funding acquisition:** Victoria Birlutiu.

**Investigation:** Victoria Birlutiu, Loredana Camelia Boicean.

**Methodology:** Victoria Birlutiu, Loredana Camelia Boicean.

**Project administration:** Victoria Birlutiu, Loredana Camelia Boicean.

**Resources:** Victoria Birlutiu, Loredana Camelia Boicean.

**Supervision:** Victoria Birlutiu, Loredana Camelia Boicean.

**Validation:** Victoria Birlutiu, Loredana Camelia Boicean.

**Visualization:** Victoria Birlutiu, Loredana Camelia Boicean.

**Writing – original draft:** Victoria Birlutiu, Loredana Camelia Boicean.

**Writing – review & editing:** Victoria Birlutiu, Loredana Camelia Boicean.

## Corrections

When originally published, the authors names were incorrect and written as Birlutiu Victoria and Boicean Loredana Camelia. They have been corrected to Victoria Birlutiu and Loredana Camelia Boicean.

## References

[1] Maurya R Bhattacharya P Dey R. Leptin functions in infectious diseases. Front Immunol 2018;9:2741. 30534129 10.3389/fimmu.2018.02741 PMC6275238

[2] Mayr FB Yende S Angus DC. Epidemiology of severe sepsis. Virulence 2014;5:04–11. 10.4161/viru.27372 PMC3916382 24335434

[3] Jacobsson S Larsson P Johansson G. Leptin independently predicts development of sepsis and its outcome. J Inflamm (Lond) 2017;14:19. 28919840 10.1186/s12950-017-0167-2 PMC5594589

[4] Momin AU Melikian N Shah AM. Leptin is an endothelial-independent vasodilator in humans with coronary artery disease: evidence for tissue specificity of leptin resistance. Eur Heart J 2006;27:2294–9. 16543250 10.1093/eurheartj/ehi831

[5] Riccieri V Stefanantoni K Vasile M. Abnormal plasma levels of different angiogenic molecules are associated with different clinical manifestations in patients with systemic sclerosis. Clin Exp Rheumatol 2011;29: 2 Suppl 65: 46–52. 21586218

[6] Pehlivan Y Onat AM Ceylan N. Serum leptin, resistin and TNF-α levels in patients with systemic sclerosis: the role of adipokines in scleroderma. Int J Rheum Dis 2012;15:374–9. 22898217 10.1111/j.1756-185X.2012.01755.x

[7] Isho F. Predictive value of serum leptin in systemic sclerosis: a novel analytic cross sectional study. La Prensa médica Mexicana 2018;104:

[8] Vernooy JH Ubags ND Brusselle GG. Leptin as regulator of pulmonary immune responses: involvement in respiratory diseases. Pulm Pharmacol Ther 2013;26:464–72. 23542720 10.1016/j.pupt.2013.03.016 PMC4122282

[9] Yousef AA Amr YM Suliman GA. The diagnostic value of serum leptin monitoring and its correlation with tumor necrosis factor-alpha in critically ill patients: a prospective observational study. Crit Care 2010;14:R33. 20230641 10.1186/cc8911 PMC2887140

[10] Bornstein SR Licinio J Tauchnitz R. Plasma leptin levels are increased in survivors of acute sepsis: associated loss of diurnal rhythm, in cortisol and leptin secretion. J Clin Endocrinol Metab 1998;83:280–3. 9435456 10.1210/jcem.83.1.4610

[11] Arnalich F López J Codoceo R. Relationship of plasma leptin to plasma cytokines and human survival in sepsis and septic shock. J Infect Dis 1999;180:908–11. 10438392 10.1086/314963

[12] Tzanela M Orfanos SE Tsirantonaki M. Leptin alterations in the course of sepsis in humans. In Vivo 2006;20:565–70. 16900791

[13] Maruna P Gurlich R Fried M. Leptin as an acute phase reactant after non-adjustable laparoscopic gastric banding. Obes Surg 2001;11:609–14. 11594104 10.1381/09608920160556814

[14] Saucillo DC Gerriets VA Sheng J. Leptin metabolically licenses T cells for activation to link nutrition and immunity. J Immunol 2014;192:136–44. 24273001 10.4049/jimmunol.1301158 PMC3872216

[15] Faggioni R Jones-Carson J Reed DA. Leptin-deficient (ob/ob) mice are protected from T cell-mediated hepatotoxicity: role of tumor necrosis factor -α and IL-18. Proc Natl Acad Sci USA 2000;97:2367–72. 10681432 10.1073/pnas.040561297 PMC15807

[16] Paz-Filho G Mastronardi C Franco CB. Leptin: molecular mechanisms, systemic pro-inflammatory effects, and clinical implications. Arq Bras Endocrinol Metabol 2012;56:597–607. 23329181 10.1590/s0004-27302012000900001

[17] Farooqi IS Matarese G Lord GM. Beneficial effects of leptin on obesity, T cell hyporesponsiveness, and neuroendocrine/metabolic dysfunction of human congenital leptin deficiency. J Clin Invest 2002;110:1093–103. 12393845 10.1172/JCI15693 PMC150795

[18] Wauman J Tavernier J. Leptin receptor signaling: pathways to leptin resistance. Front Biosci (Landmark Ed) 2011;16:2771–93. 21622208 10.2741/3885

[19] Otero M Lago R Gomez R. Towards a pro-inflammatory and immunomodulatory emerging role of leptin. Rheumatology (Oxford) 2006;45:944–50. 16720637 10.1093/rheumatology/kel157

[20] Vernooy JH Drummen NE van Suylen RJ. Enhanced pulmonary leptin expression in patients with severe COPD and asymptomatic smokers. Thorax 2009;64:26–32. 18835960 10.1136/thx.2007.085423

[21] Morash B Li A Murphy PR. Leptin gene expression in the brain and pituitary gland. Endocrinology 1999;140:5995–8. 10579368 10.1210/endo.140.12.7288

[22] Masuzaki H Ogawa Y Sagawa N. Nonadipose tissue production of leptin: leptin as a novel placenta-derived hormone in humans. Nat Med 1997;3:1029–33. 9288733 10.1038/nm0997-1029

[23] Smith-Kirwin SM O’Connor DM De Johnston J. Leptin expression in human mammary epithelial cells and breast milk. J Clin Endocrinol Metab 1998;83:1810–3. 9589698 10.1210/jcem.83.5.4952

[24] Mix H Widjaja A Jandl O. Expression of leptin and leptin receptor isoforms in the human stomach. Gut 2000;47:481–6. 10986207 10.1136/gut.47.4.481 PMC1728089

[25] Wang J Liu R Liu L. The effect of leptin on lep expression is tissue-specific and nutritionally regulated. Nat Med 1999;5:895–9. 10426312 10.1038/11335

[26] Wang J Liu R Hawkins M. A nutrient-sensing pathway regulates leptin gene expression in muscle and fat. Nature 1998;393:684–8. 9641678 10.1038/31474

[27] Zhang AJ To KK Li C. Leptin mediates the pathogenesis of severe 2009 pandemic influenza A(H1N1) infection associated with cytokine dysregulation in mice with diet-induced obesity. J Infect Dis 2013;207:1270–80. 23325916 10.1093/infdis/jit031

[28] Alti D Sambamurthy C Kalangi SK. Emergence of leptin in infection and immunity: scope and challenges in vaccines formulation. Front Cell Infect Microbiol 2018;8:147. 29868503 10.3389/fcimb.2018.00147 PMC5954041

[29] La Cava A Matarese G. The weight of leptin in immunity. Nat Rev Immunol 2004;4:371–9. 15122202 10.1038/nri1350

[30] Grinspoon S Gulick T Askari H. Serum leptin levels in women with anorexia nervosa. J Clin Endocrinol Metab 1996;81:3861–3. 8923829 10.1210/jcem.81.11.8923829

[31] Wabitsch M Jensen PB Blum WF. Insulin and cortisol promote leptin production in cultured human fat cells. Diabetes 1996;45:1435–8. 8826983 10.2337/diab.45.10.1435

[32] Machinal F Dieudonne MN Leneveu MC. In vivo and in vitro ob gene expression and leptin secretion in rat adipocytes: evidence for a regional specific regulation by sex steroid hormones. Endocrinology 1999;140:1567–74. 10098489 10.1210/endo.140.4.6617

[33] De Pergola G. The adipose tissue metabolism: role of testosterone and dehydroepiandrosterone. Int J Obes Relat Metab Disord 2000;24: Suppl. 2: S59–63. 10997611 10.1038/sj.ijo.0801280

[34] Sarraf P Frederich RC Turner EM. Multiple cytokines and acute inflammation raise mouse leptin levels: potential role in inflammatory anorexia. J Exp Med 1997;185:171–5. 8996253 10.1084/jem.185.1.171 PMC2196098

[35] Gualillo O Eiras S Lago F. Elevated serum leptin concentrations induced by experimental acute inflammation. Life Sci 2000;67:2433–41. 11065166 10.1016/s0024-3205(00)00827-4

[36] Popa C Netea MG Radstake TR. Markers of inflammation are negatively correlated with serum leptin in rheumatoid arthritis. Ann Rheum Dis 2005;64:1195–8. 15731289 10.1136/ard.2004.032243 PMC1755600

[37] Zhang HH Kumar S Barnett AH. Tumour necrosis factor-alpha exerts dual effects on human adipose leptin synthesis and release. Mol Cell Endocrinol 2000;159:79–88. 10687854 10.1016/s0303-7207(99)00194-x

[38] Al Maskari MY Alnaqdy AA. Correlation between serum leptin levels, body mass index and obesity in Omanis. Sultan Qaboos Univ Med J 2006;6:27–31. PMC3074914 21748132

[39] Tartaglia LA Dembski M Weng X. Identification and expression cloning of a leptin receptor, OB-R. Cell 1995;83:1263–71. 8548812 10.1016/0092-8674(95)90151-5

[40] Tartaglia LA. The leptin receptor. J Biol Chem 1997;272:6093–6. 9102398 10.1074/jbc.272.10.6093

[41] Myers MG Jr. Leptin receptor signaling and the regulation of mammalian physiology. Recent Prog Horm Res 2004;59:287–304. 14749507 10.1210/rp.59.1.287

[42] Wauman J De Ceuninck L Vanderroost N. RNF41 (Nrdp1) controls type 1 cytokine receptor degradation and ectodomain shedding. J Cell Sci 2011;124:921–32. 21378310 10.1242/jcs.078055 PMC3115735

[43] Uotani S Bjorbaek C Tornoe J. Functional properties of leptin receptor isoforms: internalization and degradation of leptin and ligand-induced receptor downregulation. Diabetes 1999;48:279–86. 10334302 10.2337/diabetes.48.2.279

[44] Vernooy JH Bracke KR Drummen NE. Leptin modulates innate and adaptive immune cell recruitment after cigarette smoke exposure in mice. J Immunol 2010;184:7169–77. 20488786 10.4049/jimmunol.0900963

[45] Agrawal S Gollapudi S Su H. Leptin activates human B cells to secrete TNF-alpha, IL-6, and IL-10 via JAK2/STAT3 and p38MAPK/ERK1/2 signaling pathway. J Clin Immunol 2011;31:472–8. 21243519 10.1007/s10875-010-9507-1 PMC3132280

[46] Bjorbak C Lavery HJ Bates SH. SOCS3 mediates feedback inhibition of the leptin receptor via Tyr985. J Biol Chem 2000;275:40649–57. 11018044 10.1074/jbc.M007577200

[47] Munzberg H Myers MG Jr. Molecular and anatomical determinants of central leptin resistance. Nat Neurosci 2005;8:566–70. 15856064 10.1038/nn1454

[48] Cook WS Unger RH. Protein tyrosine phosphatase 1B: a potential leptin resistance factor of obesity. Dev Cell 2002;2:385–7. 11970889 10.1016/s1534-5807(02)00158-2

[49] Zabolotny JM Bence-Hanulec KK Stricker-Krongrad A. PTP1B regulates leptin signal transduction in vivo. Dev Cell 2002;2:489–95. 11970898 10.1016/s1534-5807(02)00148-x

[50] Flak JN Myers MG Jr. Minireview: CNS mechanisms of leptin action. Mol Endocrinol 2016;30:03–12. 10.1210/me.2015-1232 PMC4695630 26484582

[51] Kelesidis T Kelesidis I Chou S. Narrative review: the role of leptin in human physiology: emerging clinical applications. Ann Intern Med 2010;152:93–100. 20083828 10.1059/0003-4819-152-2-201001190-00008 PMC2829242

[52] Klok MD Jakobsdottir S Drent ML. The role of leptin and ghrelin in the regulation of food intake and body weight in humans: a review. Obes Rev 2007;8:21–34. 17212793 10.1111/j.1467-789X.2006.00270.x

[53] Pinto S Roseberry AG Liu H. Rapid rewiring of arcuate nucleus feeding circuits by leptin. Science 2004;304:110–5. 15064421 10.1126/science.1089459

[54] Hussain Z Khan JA. Food intake regulation by leptin: mechanisms mediating gluconeogenesis and energy expenditure. Asian Pac J Trop Med 2017;10:940–4. 29111188 10.1016/j.apjtm.2017.09.003

[55] Chan JL Heist K DePaoli AM. The role of falling leptin levels in the neuroendocrine and metabolic adaptation to short-term starvation in healthy men. J Clin Invest 2003;111:1409–21. 12727933 10.1172/JCI17490 PMC154448

[56] Chan JL Matarese G Shetty GK. Differential regulation of metabolic, neuroendocrine, and immune function by leptin in humans. Proc Natl Acad Sci U S A 2006;103:8481–6. 16714386 10.1073/pnas.0505429103 PMC1482518

[57] Chan JL Williams CJ Raciti P. Leptin does not mediate short-term fasting-induced changes in growth hormone pulsatility but increases IGF-I in leptin deficiency states. J Clin Endocrinol Metab 2008;93:2819–27. 18445667 10.1210/jc.2008-0056 PMC2453057

[58] Cordero P Campion J Milagro FI. Leptin and TNF-alpha promoter methylation levels measured by MSP could predict the response to a low-calorie diet. J Physiol Biochem 2011;67:463–70. 21465273 10.1007/s13105-011-0084-4

[59] Caldefie-Chezet F Poulin A Vasson MP. Leptin regulates functional capacities of polymorphonuclear neutrophils. Free Radic Res 2003;37:809–14. 14567439 10.1080/1071576031000097526

[60] Moore SI Huffnagle GB Chen GH. Leptin modulates neutrophil phagocytosis of *Klebsiella pneumoniae*. Infect Immun 2003;71:4182–415. 12819114 10.1128/IAI.71.7.4182-4185.2003 PMC161963

[61] Loffreda S Yang SQ Lin HZ. Leptin regulates proinflammatory immune responses. Faseb J 1998;12:57–65. 9438411

[62] Santos-Alvarez J Goberna R Sanchez-Margalet V. Human leptin stimulates proliferation and activation of human circulating monocytes. Cell Immunol 1999;194:06–11. 10.1006/cimm.1999.1490 10357875

[63] Martin-Romero C Santos-Alvarez J Goberna R. Human leptin enhances activation and proliferation of human circulating T lymphocytes. Cell Immunol 2000;199:15–24. 10675271 10.1006/cimm.1999.1594

[64] Fujita Y Murakami M Ogawa Y. Leptin inhibits stress-induced apoptosis of T lymphocytes. Clin Exp Immunol 2002;128:21–6. 11982586 10.1046/j.1365-2249.2002.01797.x PMC1906378

[65] Lord GM Matarese G Howard JK. Leptin modulates the T-cell immune response and reverses starvation-induced immunosuppression. Nature 1998;394:897–901. 9732873 10.1038/29795

[66] Bruno A Chanez P Chiappara G. Does leptin play a cytokine-like role within the airways of COPD patients? Eur Respir J 2005;26:398–405. 16135719 10.1183/09031936.05.00092404

[67] Zarkesh-Esfahani H Pockley AG Wu Z. Leptin indirectly activates human Neutrophils via induction of TNF-α. J Immunol 2004;172:1809–14. 14734764 10.4049/jimmunol.172.3.1809

[68] Matarese G Moschos S Mantzoros CS. Leptin in immunology. J Immunol 2005;174:3137–42. 15749839 10.4049/jimmunol.174.6.3137

[69] Velloso LA Savino W Mansour E. Leptin action in the thymus. Ann N Y Acad Sci 2009;1153:29–34. 19236325 10.1111/j.1749-6632.2008.03973.x

[70] Da Silva SV Salama C Renovato-Martins M. Increased leptin response and inhibition of apoptosis in thymocytes of young rats offspring from protein deprived dams during lactation. PLoS One 2013;8:e64220. 23675529 10.1371/journal.pone.0064220 PMC3651239

[71] Tankersley CG O’Donnell C Daood MJ. Leptin attenuates respiratory complications associated with the obese phenotype. J Appl Physiol 1998;85:2261–9. 9843551 10.1152/jappl.1998.85.6.2261

[72] O’Donnell CP Schaub CD Haines AS. Leptin prevents respiratory depression in obesity. Am J Respir Crit Care Med 1999;159:1477–84. 10228114 10.1164/ajrccm.159.5.9809025

[73] Piitulainen E Areberg J Linden M. Nutritional status and muscle strength in patients with emphysema and severe alpha(1)antitrypsin deficiency. Chest 2002;122:1240–6. 12377848 10.1378/chest.122.4.1240

[74] Schattner M. Enteral nutritional support of the patient with cancer: route and role. J Clin Gastroenterol 2003;36:297–302. 12642734 10.1097/00004836-200304000-00004

[75] Jedrychowski W Maugeri U Flak E. Predisposition to acute respiratory infections among overweight preadolescent children: an epidemiologic study in Poland. Public Health 1998;112:189–95. 9629027 10.1038/sj.ph.1900438

[76] Baik I Curhan GC Rimm EB. A prospective study of age and lifestyle factors in relation to community-acquired pneumonia in US men and women. Arch Intern Med 2000;160:3082–8. 11074737 10.1001/archinte.160.20.3082

[77] Van Kerkhove MD Vandemaele KA Shinde V. Risk factors for severe outcomes following 2009 influenza A (H1N1) infection: a global pooled analysis. PLoS Med 2011;8:e1001053. 21750667 10.1371/journal.pmed.1001053 PMC3130021

[78] Kwong JC Campitelli MA Rosella LC. Obesity and respiratory hospitalizations during influenza seasons in Ontario, Canada: a cohort study. Clin Infect Dis 2011;53:413–21. 21844024 10.1093/cid/cir442 PMC3156143

[79] Díez ML Santolaria F Tejera A. Serum leptin levels in community acquired pneumonia (CAP) are related to nutritional status and not to acute phase reaction. Cytokine 2008;42:156–60. 18396058 10.1016/j.cyto.2008.02.013

[80] Parmentier-Decrucq E Nseir S Makris D. Accuracy of leptin serum level in diagnosing ventilator-associated pneumonia: a case-control study. Minerva Anestesiol 2014;80:39–47. 24107832

[81] Behnes M Brueckmann M Lang S. Alterations of leptin in the course of inflammation and severe sepsis. BMC Infect Dis 2012;12:217. 22973876 10.1186/1471-2334-12-217 PMC3462137

[82] Mancuso P Gottschalk A Phare SM. Leptin-deficient mice exhibit impaired host defense in Gram-negative pneumonia. J Immunol 2002;168:4018–24. 11937559 10.4049/jimmunol.168.8.4018

[83] Hsu A Aronoff DM Phipps J. Leptin improves pulmonary bacterial clearance and survival in ob/ob mice during pneumococcal pneumonia. Clin Exp Immunol 2007;150:332–9. 17822444 10.1111/j.1365-2249.2007.03491.x PMC2219341

[84] Cederholm T Lindgren JA Palmblad J. Impaired leukotriene C4 generation in granulocytes from protein-energy malnourished chronically ill elderly. J Intern Med 2000;247:715–22. 10886494 10.1046/j.1365-2796.2000.00691.x

[85] Moore SI Huffnagle GB Chen GH. Leptin modulates neutrophil phagocytosis of Klebsiella pneumoniae. Infect Immun 2003;71:4182–5. 12819114 10.1128/IAI.71.7.4182-4185.2003 PMC161963

[86] Mancuso P Peters-Golden M Goel D. Disruption of leptin receptor-STAT3 signaling enhances leukotriene production and pulmonary host defense against pneumococcal pneumonia. J Immunol 2011;186:1081–90. 21148797 10.4049/jimmunol.1001470 PMC3133444

[87] Mancuso P Myers MG Jr Goel D. Ablation of leptin receptor-mediated ERK activation impairs host defense against gram-negative pneumonia. J Immunol 2012;189:867–75. 22685316 10.4049/jimmunol.1200465 PMC3392451

[88] Ordway D Henao-Tamayo M Smith E. Animal model of Mycobacterium abscessus lung infection. J Leukoc Biol 2008;83:1502–11. 18310351 10.1189/jlb.1007696

[89] Easterbrook JD Dunfee RL Schwartzman LM. Obese mice have increased morbidity and mortality compared to non-obese mice during infection with the 2009 pandemic H1N1 influenza virus. Influenza Other Respi Virus 2011;5:418–25. 10.1111/j.1750-2659.2011.00254.x PMC3175349 21668672

[90] Lin S Thomas TC Storlien LH. Development of high fat diet-induced obesity and leptin resistance in C57Bl/6J mice. Int J Obes Relat Metab Disord 2000;24:639–46. 10849588 10.1038/sj.ijo.0801209

[91] Karlsson EA Sheridan PA Beck MA. Diet-induced obesity impairs the T cell memory response to influenza virus infection. J Immunol 2010;184:3127–33. 20173021 10.4049/jimmunol.0903220

[92] Karlsson EA Sheridan PA Beck MA. Diet-induced obesity in mice reduces the maintenance of influenza-specific CD8+ memory T cells. J Nutr 2010;140:1691–7. 20592105 10.3945/jn.110.123653 PMC2924599

[93] Smith AG Sheridan PA Harp JB. Diet-induced obese mice have increased mortality and altered immune responses when infected with influenza virus. J Nutr 2007;137:1236–43. 17449587 10.1093/jn/137.5.1236

[94] Smith AG Sheridan PA Tseng RJ. Selective impairment in dendritic cell function and altered antigen-specific CD8+ T-cell responses in diet induced obese mice infected with influenza virus. Immunology 2009;126:268–79. 18754811 10.1111/j.1365-2567.2008.02895.x PMC2632688

[95] Morgan OW Bramley A Fowlkes A. Morbid obesity as a risk factor for hospitalization and death due to 2009 pandemic influenza A(H1N1) disease. PLoS One 2010;5:e9694. 20300571 10.1371/journal.pone.0009694 PMC2837749

[96] Gruzdeva O Borodkina D Uchasova E. Leptin resistance: underlying mechanisms and diagnosis. Diabetes Metab Syndr Obes 2019;12:191–8. 30774404 10.2147/DMSO.S182406 PMC6354688

[97] Caballero B. The global epidemic of obesity: an overview. Epidemiol Rev 2007;29:01–5. 10.1093/epirev/mxm012 17569676

[98] Prescott HC Chang VW O’Brien JM Jr. Obesity and 1year outcomes in older Americans with severe sepsis. Crit Care Med 2014;42:1766–74. 24717466 10.1097/CCM.0000000000000336 PMC4205159

[99] Wong GW Wang J Hug C. A family of Acrp30/adiponectin structural and functional paralogs. Proc Natl Acad Sci U S A 2004;101:10302–7. 15231994 10.1073/pnas.0403760101 PMC478567

[100] Martin SS Qasim A Reilly MP. Leptin resistance: a possible interface of inflammation and metabolism in obesity-related cardiovascular disease. J Am Coll Cardiol 2008;52:1201–10. 18926322 10.1016/j.jacc.2008.05.060 PMC4556270

[101] Ntaios G Gatselis NK Makaritsis K. Adipokines as mediators of endothelial function and atherosclerosis. Atherosclerosis 2013;227:216–21. 23332774 10.1016/j.atherosclerosis.2012.12.029

[102] Ouchi N Parker JL Lugus JJ. Adipokines in inflammation and metabolic disease. Nat Rev Immunol 2011;11:85–97. 21252989 10.1038/nri2921 PMC3518031

[103] Shapiro NI Schuetz P Yano K. The association of endothelial cell signaling, severity of illness, and organ dysfunction in sepsis. Crit Care 2010;14:R182. 20942957 10.1186/cc9290 PMC3219288

[104] Shapiro NI Khankin EV Van Meurs M. Leptin exacerbates sepsis-mediated morbidity and mortality. J Immunol 2010;185:517–24. 20519646 10.4049/jimmunol.0903975 PMC3997057

[105] Shufen L Xi L. Leptin in normal physiology and leptin resistance. Sci Bull 2016;61:1480–8.

[106] Dayakar A Chandrasekaran S Veronica J. Leptin induces the phagocytosis and protective immune response in Leishmania donovani infected THP1 cell line and human PBMCs. Exp Parasitol 2016;160:54–9. 26688099 10.1016/j.exppara.2015.12.002

[107] Goldberg EL Romero-Aleshire MJ Renkema KR. Lifespan-extending caloric restriction or mTOR inhibition impair adaptive immunity of old mice by distinct mechanisms. Aging Cell 2015;14:130–8. 25424641 10.1111/acel.12280 PMC4326902

[108] Iyer SS Chatraw JH Tan WG. Protein-energy malnutrition impairs homeostatic proliferation of memory CD8 T cells. J Immunol 2012;188:77–84. 22116826 10.4049/jimmunol.1004027 PMC3244573

[109] Madan R Guo X Naylor C. Role of leptinmediated colonic inflammation in defense against Clostridium difficile colitis. Infect Immun 2014;82:341–9. 24166957 10.1128/IAI.00972-13 PMC3911837

[110] Blüher S Mantzoros CS. Leptin in humans: lessons from translational research. Am J Clin Nutr 2009;89:991S–7S. 19176740 10.3945/ajcn.2008.26788E PMC2667664

[111] McCowen K Malhotra A Bistrian BR. Stress-inducedhyperglycemia. In: endocrine and metabolic dysfunction syndromes in the critically ill. Crit Care Clinics 2001;17:107–24. 10.1016/s0749-0704(05)70154-8 11219223

[112] Langouche L Van den Berghe G. Hypothalamic-pituitary hormones during critical illness: a dynamic neuroendocrine response. Handb Clin Neurol 2014;124:115–26. 25248583 10.1016/B978-0-444-59602-4.00008-3

[113] Cornejo MP Hentges ST Maliqueo M. Neuroendocrine regulation of metabolism. J Neuroendocrinol 2016;28:10.1111/jne.12395. 10.1111/jne.12395 PMC4956544 27114114

[114] Téblick A Langouche L Van den Berghe G. Anterior pituitary function in critical illness. Endocr Connect 2019;8:131–43. 10.1530/EC-19-0318 PMC6709544 31340197

[115] Singer M Deutschman CS Seymour CW. The third international consensus definitions for sepsis and septic shock (sepsis-3). JAMA 2016;315:801–10. 26903338 10.1001/jama.2016.0287 PMC4968574

[116] Vincent JL Moreno R Takala J. The SOFA (sepsis-related organ failure assessment) score to describe organ dysfunction/failure. On behalf of the working group on sepsis-related problems of the European Society of Intensive Care Medicine. Intensive Care Med 1996;22:707–10. 8844239 10.1007/BF01709751

[117] Pollard S Edwin SB Alaniz C. Vasopressor and Inotropic Management Of Patients With Septic Shock. P T 2015;40:438–50. 26185405 PMC4495871

[118] Vallejos A Olivares P Varela D. Preventive leptin administration protects against sepsis through improving hypotension, tachycardia, oxidative stress burst, multiple organ dysfunction, and increasing survival. Front Physiol 2018;9:1800. 30618812 10.3389/fphys.2018.01800 PMC6299116

[119] Takahashi H Tsuda Y Takeuchi D. Influence of systemic inflammatory response syndrome on host resistance against bacterial infections. Crit Care Med 2004;32:1879–85. 15343016 10.1097/01.ccm.0000139606.34631.61

[120] Tschöp J Nogueiras R Haas-Lockie S. CNS leptin action modulates immune response and survival in sepsis. J Neurosci 2010;30:6036–47. 20427662 10.1523/JNEUROSCI.4875-09.2010 PMC2868384

